# Supplementary material for: Are carbon emissions trading and green financial instruments synergistic? -Comprehensive quantitative research based on content analysis
Source: PLoS One. 2024 Mar 7;19(3):e0298601. doi: 10.1371/journal.pone.0298601 (PMC10919601; doi:10.1371/journal.pone.0298601)
Supplement: S1 Data — (ZIP) [file pone.0298601.s001.zip › Data/地级市Z_F工作报告.zip!/┴╔─■/╠·┴δ╩╨/└┤╫╘╣1⁄2╓┌║┼í╛┬φ┐╦╩2╛▌═°í┐_2014.docx]

各位代表：

现在，我代表市人民政府向大会报告工作，请予审议，并请市政协委员和列席同志提出意见。

一、2013年工作回顾

刚刚过去的2013年，是我们战胜各种困难、奋勇闯过难关的一年，是锐意推进改革、大胆进行创新的一年，是埋头苦干实干、加倍付出艰辛的一年。我们在省委、省政府和市委的正确领导下，发展铁岭经济，提高百姓福祉，各项工作全面提速、提质、提效，全市经济社会发展取得新的成绩。

—— 国民经济平稳发展。预计，全市地区生产总值实现1050亿元，增长8%，三次产业结构比为18：52：30；公共财政预算收入103.6亿元，增长12%（可比口径）；固定资产投资1050亿元，增长16%；规模以上工业增加值增长7%；社会消费品零售总额348亿元，增长13%；城镇居民人均可支配收入20550元，增长10.5%；农民人均纯收入11837元，增长12%。

—— 对外开放取得新成果。引进域外投资项目808个，到位内资960亿元。实际利用外资、外贸出口均突破5亿美元，辽宁石油机械公司国际工程总承包实现重大突破。

具有国际国内领先技术的德国莱尼线束、士弗瑞轮毂等一批项目落地开工，九三粮油三期、辉山乳业全产业链、航天万源、寿光果蔬贸易城、兴东纸业等项目建设速度加快，其中516个建成投产。斯迈特电梯、伯爵油漆等8个工业地产项目开工建设，完成投资48亿元，走在全省前列。首届两岸和谐家庭合唱节成功举办，台湾工业园建设取得新进展。保税物流中心申报工作开始启动。

我市以全国第三名的成绩，成功入选国家节能减排财政政策综合示范城市。调兵山市获批为国家循环经济示范县。银州区铁西老工业区搬迁改造方案通过国家验收。开发区晋升为国家级开发区，结束了我市没有国家级开发区的历史。

—— 工业经济发展水平进一步提升。产业集群建设取得新进展，实现销售收入1800亿元，协作配套率达到30%。专用车生产基地被确定为国家新型工业化产业示范基地，采取档案封存、绩效管理模式组建了招商管理局，骨干企业进一步做大做强，东北三省巡展取得丰硕成果。换热设备产业基地与汽车零部件产业园、生活配套区同步建设，形成了产城互动新格局。全国首家工业超市东北煤机物流中心投入运营，煤机装备产业集群获批为省新型工业化产业示范基地。起重机械产业集群被确定为国家火炬计划特色产业基地，卫华起重机超市当年建设当年投入运营。石油机械产业集群跻身省重点产业集群行列。生命健康、工程机械、汽车零部件等产业集群建设取得新进展。橡塑、阀门等传统产业发展迈出新步伐。

双百工程取得新成效，盘活闲置土地2604亩，盘活停产半停产企业24家，完成朗克药业、东升冶金等5户企业并购，实施技改项目100项，销售收入10亿元以上企业发展到26户。

新增高新技术企业12户，特阀公司被认定为国家火炬计划重点高新技术企业，全市高新技术产品产值实现588亿元。中科院国家技术转移中心铁岭中心挂牌，专用车生产基地和生命健康产业集群两家院士工作站启动，亚洲最大的舒瑞普换热设备研发中心落户昌图，高温大口径抽油泵等12个新产品、新材料填补国内空白。我市再获全国科技进步先进市，5个县（市）全部被评为全国科技进步先进县。

—— 城镇化迈出新步伐。按照以人为本、让利于民、产城互动、绿色发展的原则，创造性地实施了以单户城镇化为主的新型城镇化，探索出解决资金、土地、人口集聚等问题的新路径，已有9086户农民在凡河城区选房，其中2447户选现房，7个村屯、2个社区实现了整村整屯进城。全市房地产新开发面积1980万平方米。沈铁同城化上升为全省发展战略。

凡河城区功能进一步完善，22个便民商业网点开业，东北民俗文化园、兴隆大船超市、综合客运枢纽站等投入运营，76家事业单位迁入。市医院凡河院区引进了高端医疗设备，正式对外开诊，并为全市教师进行了免费体检。两环一路公交开通，新三线公路一期工程竣工通车。组建了社会事业管理局和2个社区，设置了便民服务大厅，全面履行社会管理职能。以确保百姓温暖过冬为目标，落实供热补贴政策和监管措施，百姓满意率全面提升。

进一步理顺了银州区管理体制，靠山屯棚改回迁楼竣工，沙子沟棚改工程开工建设，改造街路97条、40万平方米，新增集中供热面积300万平方米，龙山体育公园、铁西汇工休闲广场建成并投入使用，站前广场改造工程获中国园艺杯金奖。县城建设取得新进展，开原市、调兵山市、昌图县被确定为全省城镇化重点县。行政区划调整第一阶段顺利完成。

在全省率先实施新居住证管理制度，12万农业转移人口有序实现市民化。全市城镇化率达到50.2%，提高近4个百分点。

—— 农业农村经济健康发展。粮食生产实现九连丰，总产量超过87亿斤，昌图县被评为全国粮食生产标兵县。

蔬菜、榛子产量分别达到550万吨和4万吨，成功举办铁岭榛子（沈阳）展销会，开辟了优质农产品走向域外市场的新路径。我市获全国林业产业发展突出贡献奖，开原市被授予国家重点花木良种示范基地。新建标准化规模养殖小区200个，新增国家菜篮子基地16个，畜牧业产值突破300亿元，开原市、昌图县被确定为全省现代畜牧示范区。

新增农民合作社标准示范社50家，家庭农场发展到1096个，流转土地162万亩。农业产业化产值实现1260亿元。

实施农建骨干工程393项，完成节水滴灌50万亩。农业机械化水平达到70%，水稻生产实现全程机械化。气象服务和应对自然灾害能力明显提升，战胜了“8?16”特大洪灾。

新增绿色食品、无公害农产品认证18个，“曙光”被评为中国驰名商标。铁岭县农业科技园区获批为国家农业科技园区，西丰县被授予国家级鹿产品出口质量安全示范区。

—— 现代服务业加快发展。开原大润发、调兵山新时代开业，星悦南岸、天力婚庆城、昌图禧徕乐等项目进展顺利。农资物流园、海鑫汽车城进入招商运营阶段，东北物流城市场规模进一步扩大。新增省级服务业集聚区2个、双百工程重点项目6个，东北参茸中草药材市场获批为全国电子商务示范企业。

打造了万亩花海，辽河博物馆交付使用，新浪潮水世界、汽车嘉年华、温泉小镇等项目取得积极进展，成功举办首届中国铁岭荷花文化旅游节，全市旅游业总收入实现149亿元。

会展业快速发展，专用车博览会被评为辽宁省十大品牌展会。形成“夜经济”特色街区和夜市19个，成为消费新亮点。

—— 生态文明建设取得新成效。青山、碧水、蓝天、“美丽铁岭”城乡环境综合整治工程扎实推进，万元生产总值能耗下降5.15%，四项主要污染物排放量平均减少1.5%。市级机关事务管理局被评为全国节约型公共机构示范单位。

完成小开荒清退、超坡地还林、围栏封育74万亩，植树造林26万亩，西丰县“两退一围”经验在全省推广。

国家重大水专项取得阶段性成果，辽河铁岭段水质全年优于Ⅳ类标准。莲花湿地、凡河湿地纳入全国湿地保护“十二五”规划，开原白鹭洲晋升省级自然保护区。

空气环境质量监测达标率达到97.8%，列全省第三位。

新修村屯道路1916公里，种植绿化树木416万株，城市垃圾实现日产日清，40%以上的农村垃圾实现户投放、村收集、镇转运、县处理，铁岭县农村环境整治工作经验在全省推广。

—— 发展保障能力得到增强。城乡建设用地增减挂钩工作实现历史性突破，获批周转指标10平方公里，列全省第一位。我市被评为全国国土资源节约集约模范市，获用地指标奖励1平方公里。成立市规划土地委员会，对建设用地进行统一管理，实现土地出让金收入62亿元。

向上争取各类无偿资金133亿元。多渠道筹措资金，确保政府债务按时还本付息，维护了政府信用，实现了良性循环。

金融生态环境明显改善，第一家域外股份制商业银行浦发银行开业，英大、安邦、安华3家保险机构落户。金融机构存、贷款余额分别实现910亿元和701亿元，新增贷款87亿元。

安邦农业贷款担保业务签订协议。爱华照明、特阀公司、银捷机械分别在上海和辽宁股权交易市场挂牌。

—— 百姓福祉进一步提高。投入资金103亿元，重点实施了就业社保、外墙保温、农村危房改造、教学条件改善、安全饮水等十大民生工程。

安置实名制就业40575人，城镇登记失业率控制在2.8%以内，零就业家庭实现动态为零。城镇五险参保人数达193万人次，新农保参保率达到96%，我市被评为国家金保工程示范城市。

城乡低保年保障标准分别提高420元和240元，集中和分散五保户年供养标准分别提高400元和300元。市残疾人康复中心投入运营。市殡仪服务中心交付使用。

铁岭县农村学前教育连锁办园模式在全省推广，沈阳南宁幼儿园分园正式开园。铁岭高中获北大招生推荐资格，成立了“周恩来班”。铁岭师专、辽宁工程职业学院对外合作办学迈出新步伐，辽宁职业学院被列入国家示范性职业院校建设计划。我市荣获全国教育改革创新特别贡献奖。

新农合覆盖全市农业人口，筹资标准提高到350元。人均基本公共卫生服务标准提高到30元。人口计生工作取得新成绩。

成功举办首届魏燮均杯书法作品展、高其佩杯美术作品展、全国魏燮均学术研讨会，铁岭欢歌群众广场文化活动成为全省品牌，银冈书院等4家单位被评为国家级文物保护单位，昌图县被命名为中国书法之乡。圆满承办全国十二运铁岭赛区赛事，市体校成为国家高水平体育后备人才基地。

新增保障性安居工程6257户，修复水毁住房4600户。新建农村社区80个，城市社区十分钟服务圈建设步伐加快。

—— 社会保持和谐稳定。坚持市县领导接访包案制度，成立全省首家平安文化教育基地，基层法律援助工作走在全国前 列，连续两年荣获全国见义勇为优秀组织奖，群众安全感和满意度列全省第一名。国防动员和应急管理工作得到加强，安全生产形势总体平稳，食品药品安全监管能力进一步提升。

整合877部公开电话，建立了12345政府服务热线。公共资源实现统一集中交易，批管分离工作成效显著，三维可视化电子政务大厅投入运行，乡镇公共行政服务中心实现全覆盖。政务公开迈出新步伐，绩效管理和政务督查工作得到加强，依法行政水平进一步提升。

全年办理人大议案、人大代表建议和政协提案563件，见面率、办复率和满意率均达到100%。

民族、宗教、审计、外事、侨务、规划、综合执法、统计、人防、物价、地震、国家安全、国税、地税、质监、武警、消防、邮政、通信、烟草、出入境检验检疫、供电、档案、政务接待、妇女儿童等工作也都取得了新成绩。

各位代表：取得这些成绩，得益于省委、省政府和市委的科学决策、正确领导，得益于市人大、市政协的有效监督、鼎力支持，得益于全市人民的同心协力、团结奋斗。在此，我代表市人民政府，向全市各族人民，向市人大代表、政协委员、各民主党派、工商联、无党派人士和人民团体，向中省直驻铁单位、解放军驻铁部队和武警官兵，向所有关心支持铁岭发展的广大客商和海内外朋友，表示衷心的感谢和崇高的敬意！

在看到成绩的同时，也要清醒地看到目前存在的问题。经济总量还不大，财政收入质量不高，外贸依存度低，缺少安身立命的大项目，发展的内生动力不足。经济结构不尽合理，农业优势还没有充分发挥，服务业发展相对滞后。工业经济实力不强，处于产业链低端，产品附加值偏低。城镇化水平还有很大差距，城乡环境整治任务繁重，民生工程欠账较多。政府职能转变还不到位，软环境建设仍有不尽人意之处。这些问题已经引起我们高度重视，并将采取有力措施认真加以解决。

二、2014年的主要工作

今年是贯彻落实十八届三中全会精神、完成“十二五”规划任务目标的关键之年，做好各项工作具有十分重要的意义。政府工作的指导思想是：坚持稳中求进、稳中求优的总基调，把握发展的问题导向、民生导向、环境导向、安全导向，以项目建设为核心，以对外开放、改革创新为牵动，着力激发市场活力，着力转方式调结构，发展铁岭经济，提高百姓福祉，各项工作全面提速、提质、提效，全面开创幸福美好新铁岭建设的新局面。

主要预期目标是：地区生产总值增长9.5%，公共财政预算收入增长10%，固定资产投资增长19%，规模以上工业增加值增长10%，社会消费品零售总额增长13.5%，实际利用外资、外贸出口分别增长15%，城镇居民人均可支配收入、农民人均纯收入分别增长12%。重点抓好以下10项工作：

（一）全力提高对外开放水平，着力引进安身立命大项目

瞄准国内外500强和行业领军企业，依托资源和产业优势，针对重点地区开展招商活动。各级党政主要领导要带队招商，进一步强化市级领导包保和挂牌督办责任制，每月召开一次重大项目推进办公会，做到重策划、精包装、强推介、求实效。选派优秀干部到北京、上海、深圳等地挂职，引进和培养市场化、专业化的复合型招商人才。

更加注重项目的附加值和投资回报率，围绕发展高端产业链，推动招商引资向招商选资转变，着力引进建设投资强度大、发展潜力大、对税收和就业贡献率高的大项目。重点抓好辉山乳业、平安养生小镇、长江线缆等投资50亿元以上项目，各县（市）区、开发区和高新区分别引进一个投资30亿元以上项目，全年到位内资960亿元以上，实际利用外资6.3亿美元以上。

加强对台经贸文化交流与合作，促进台湾工业园发展。全力抓好保税物流中心建设，力争新建2个省级外贸出口基地，鼓励企业走出去开拓国际市场，外贸出口实现7.2亿美元以上。

（二）提高产业集群发展水平，促进工业提质增效升级

8个省级产业集群分别建设一个工业超市，协作配套率力争达到40%，办好博览会或展洽会，销售收入增长20%以上。

举全市之力发展专用车生产基地，进一步创新运营机制，搭建电子商务、统一采购、研发检测、投融资四大平台，培育陆平、中欧、际华等10户骨干企业，打造城建装备、商务车、新能源汽车、LNG罐车四大板块，早日实现500亿元产值目标。

换热设备产业基地抓好远大成套装置、舒瑞普研发中心等项目，加快产城融合发展步伐，叫响中国热谷品牌。

起重机械产业集群抓好德马格、斯迈特电梯等项目，检验检测中心投入使用，完善产业链条，打造中国起重机之都。

煤机装备产业集群围绕东北煤机物流中心，引进上下游配套企业，尽快实现不出调兵山就能装备一座矿山的目标。

石油机械产业集群抓好辽宁石油机械工业地产、国际工程总承包项目，打造具有国际影响力的石油装备生产基地。

生命健康产业集群抓好辉山乳业全产业链、贵州茅台等项目，积极创建国家级可持续发展试验区。

汽车零部件产业集群依托区位优势，主动承接沈阳和长春产业转移，重点抓好产业园和物流园建设，尽快做大规模。

工程机械产业集群以再制造业大市场为突破口，着力提高科技贡献率，建设东北工程机械产业服务基地。

大力发展工业地产和混合所有制经济。沿新三线公路规划建设新的工业园区和新市镇，带动铁岭西部地区加快发展。

深入开展双百工程，强化兜底服务，实施工业项目和技改项目各100项，销售收入10亿元以上企业发展到35户，九三粮油、昌图曙光力争突破100亿元。

实施以企业为主体的创新战略，新增高新技术企业10户，开展产学研合作50项，开发新产品450项，“铁阀”争创国家名牌。全市高新技术产品产值实现710亿元，增长21%。

（三）坚持以人为本，加快推进新型城镇化

以凡河城区为核心，坚持以人为本、让利于民、产城互动、绿色发展的原则，有序推进组团式城镇化、单户城镇化，完善新居住证管理制度，加快农业转移人口市民化步伐。

启动以新台子镇为先导的沈铁新区建设，实施万泉河开发工程，沈铁二号公路全线贯通，积极推进沈铁三号公路和孤懿线辽河特大桥开工建设，加快推进沈铁同城化进程。

进一步完善凡河城区功能，大力发展劳动密集型、无污染产业，积极推进新浪潮水世界、耀华国际学校、兴隆综合体、官台新苑、市政花园广场等项目建设，加快人口集聚步伐。

加大多元化投资力度，合理开发城市地下空间，实施道路改造、园林绿化等工程，改善银州区人居环境。

加快推进行政区划调整。进一步理顺城区管理体制，继续向城区下放权力，做大做强城区经济。选择重点镇开展扩权强镇试点，促进城乡一体化发展，全市城镇化率提高到53%以上。

结合新型城镇化，新开发房地产面积2286万平方米。物业管理覆盖率达到80%以上，管理水平进一步提高。

开工建设光荣街南段、新三线公路二期工程，修建村内道路370公里，做好沈四高速公路拓宽改造工程前期工作。

（四）加快构建新型农业经营体系，推进农业产业化进程

全力抓好“三农”工作，让农业强起来、农村美起来、农民富起来。高举全域绿色农业的旗帜，调动好、保护好农民和粮食主产区两个积极性，从源头上确保粮食和农产品安全，提高农业附加值和贡献率，打造全国闻名的优质农产品生产基地。

继续实施百亿斤粮食产能工程，完成节水滴灌44万亩，新建新型农户储粮仓3000套，提高农业技术装备水平和抗御气象灾害能力，全力夺取粮食大丰收。

新建标准化畜牧小区200个，建设全省生猪、肉牛产业第一市，实现畜牧业产值350亿元。开发以榛子、寒富苹果、彩色苗木为主的经济林产业带，争当全省排头兵。

充分挖掘农村土地资源优势，构建以家庭经营为基础、合作组织为纽带、社会化服务为支撑的现代农业经营体系，探索建立农村土地交易平台和流转市场，推进规模化、集约化经营。新增农民合作社标准示范社50家，全市流转土地180万亩以上。

大力发展农产品深加工业，重点建设铁岭县国家农科园，推进益海嘉里、10万吨秸秆乙醇、昌图农高园等项目，规模以上龙头企业实现销售收入1300亿元。

做大做强县域经济，继续举全市之力加快西丰发展，支持昌图扩权强县，提高一县一业和县城建设水平，壮大财政实力。

（五）大力发展现代服务业，不断改善经济结构

以稳消费、促销费为目标，努力扩大消费需求，本地消费对税收的贡献率提高到10%。

搞活东北物流城，抓好苏宁云商综合体、华强综合体等重点项目，力争星悦南岸商贸商务区、莲花湿地旅游集聚区、开原市中心商贸区晋升省级服务业集聚区。

依托自然资源和满族历史文化，发展以“一区四带”为主体的旅游产业。抓好万亩花海、千顷湿地、百里荷塘、十里天水河旅游景观工程，大力开发亲水娱乐、餐饮住宿等配套项目，加快推进辽河旅游、沟域旅游、温泉旅游，办好第二届中国铁岭荷花文化旅游节、第三届满族颁金节，打造“北方水城、快乐铁岭”品牌。全市旅游业总收入实现180亿元，清河区争创国家级生态旅游示范区。

推进生产性服务业与制造业分离，培育土地、金融、技术等要素市场，大力发展电子商务和会展经济。

继续实施万村千乡市场工程和新网工程，构建农村流通体系。大力开展农产品外埠推介活动，推动铁岭农产品走出去。积极引导本地消费，在全社会形成消费家乡产品、我爱我家的浓厚氛围。

（六）加强生态文明建设，全面提升人居环境质量

设立绿色产业发展专项资金，重点实施50万平方米绿色建筑、60万吨柴河引水工程、14兆瓦垃圾发电厂等37个节能减排项目，争创国家生态文明示范市和国家环保模范城市。

加强山区生态治理和保护，抓好平原造林、镇村绿化和矿山复绿工作，完成植树造林35万亩，绿化村屯290个，建设省级绿化模范试点村20个，新增省级生态县1个、生态乡镇10个。

进一步巩固辽河治理成果，加快辽河生态文明示范区建设，力争率先建成生态带、旅游带、城镇带。

推进高效供热和气化铁岭工作。建设农村户用沼气池500户以上、生物质成型燃料炊事采暖炉1000户以上。

按照属地管理、重心下移、条块结合、以块为主的原则，坚持不懈地抓好“美丽铁岭”城乡环境综合整治，努力实现全方位、全覆盖、全域化、无死角的“三全一无”管理目标。

（七）大胆创新体制机制，进一步增强发展保障能力

强化财税收入征管，逐步提高财税收入质量。加强预算管理，优化支出结构，防范政府性债务风险。

积极开展城乡建设用地增减挂钩，力争获批周转指标10平方公里以上。开展闲置和低效用地清查行动，推进节约集约用地。加大经营城市力度，力争实现土地出让金收入60亿元。

建设金融广场，推进盛京银行、兴业银行等金融机构和金融服务机构入驻，抓好农商行组建工作，建立覆盖全市的融资项目网络数据库，完善金融市场体系，新增贷款90亿元以上。提高直接融资比重，重点培育2—3家企业上市。积极争创国家和省级试点，向上争取无偿资金150亿元。通过政府购买服务、特许经营等方式，鼓励社会资本参与城市公用设施投资运营。

实施积极的人才战略，建设学习型城市，促进人的全面发展，建立推动可持续发展、长远发展的智力支撑体系。开展好第三次经济普查，为全市发展提供科学的决策依据。

（八）着力保障和改善民生，让改革发展成果更多更公平惠及百姓

加大投入力度，围绕百姓关心关注的问题，重点抓好民生十件实事。1.新建维修改造街路100条。2.继续改造集中连片棚户区，新增保障性安居工程21497户。3.对500户贫困残疾人住房进行无障碍改造。4.实施莲花湿地三期工程。5．新增实名制就业5万人以上。6.实施空气质量改善和垃圾处理场站建设工程。7.建设铁岭大剧院、美术馆和10处历史名人故居（纪念馆）。8.为1000个村屯安装路灯。9.积极发展居家养老等社会养老事业。10.实施暖房子工程300万平方米，中心城区集中供热率提高3个百分点。

重点抓好高校毕业生、进城农民、困难群体和新增劳动力就业工作，城镇登记失业率控制在3.8%以内。城镇五险参保人数达到200万人次，城镇居民社会养老保险参保率达到85%以上。完善城乡救助体系，适时提高城乡困难群众救助供养标准，对贫困家庭先天性心脏病患者进行救助，开展福彩助学活动。新建农村饮水安全工程70处，解决6万人饮水不安全问题。

（九）加快社会事业改革与发展，提高社会治理水平

继续开展教育年活动，实施连锁幼儿园建设工程，为771辆校车安装卫星定位装置，清河区教育强区通过省验收。建设青少年素质教育基地，推动学校对外交流与合作，推进铁岭师专升本，支持辽宁职业学院创建国家示范性高职院校。

实施健康促进工程，广泛开展爱国卫生运动，提高新农合受益水平。抓好乡村卫生服务一体化管理和公立医院改革，完成市县食品药品监管、卫生计生等体制改革工作。

组织好纳兰性德杯诗词大赛、端木蕻良杯文学大赛、台湾纸风车剧团展演等文化活动，申办东北二人转文化艺术节。发挥铁岭工笔画、指画、剪纸优势，培育一批特色文化村。继续开展全民健身活动，积极组织参加省十二运。

深入推进平安铁岭、法治铁岭建设，切实增强应急管理能力，完善安全生产责任体系和监管责任制。加强信访工作，完善政府服务热线运行机制，及时解决百姓合理诉求。构建“三位一体”大调解体系，做好社区矫正工作。新建农村社区80个。

落实党的宗教政策，积极推进民族团结进步事业。加强国防动员和后备力量建设，巩固双拥共建成果。培育和践行社会主义核心价值观，加快构建社会诚信体系，争创全国文明城市。

（十）切实转变政府职能，建设法治政府和服务型政府

自觉接受市人大及其常委会的法律监督、工作监督，定期向人大报告工作，认真落实市人大及其常委会的决议和决定。积极支持市政协履行政治协商、民主监督和参政议政职能。坚持定期向各民主党派、工商联和无党派人士通报工作。认真办理好人大议案、人大代表建议和政协提案。

依法履行政府职能，健全依法行政责任体系和各项监督制度，进一步简政放权，加大政务公开力度，科学开展绩效管理，强化政务督查，创造稳定、透明、公平的发展环境。

认真贯彻落实中央八项规定，开展好党的群众路线教育实践活动，坚决遏制形式主义、官僚主义、享乐主义和奢靡之风，做到讲诚信、懂规矩、守纪律。加强五大系统建设，巩固完善覆盖全社会的惩防体系，建设廉洁政府。牢固树立过紧日子思想，大力压缩三公经费，形成勤俭节约风气。

倡导思维顶天、工作立地的理念，发扬事不过夜、事无巨细、事必躬亲的工作作风，以钉钉子的精神，以功成不必在我的境界，多做打基础、利长远的工作，多做推功揽过的事情，积极营造干事创业的浓厚氛围。

各位代表，发展铁岭经济时不我待，提高百姓福祉任重道远。让我们紧密团结在以习近平同志为核心的党中央周围，高举中国特色社会主义伟大旗帜，在中共铁岭市委的正确领导下，开拓创新，埋头苦干，为建设幸福美好新铁岭而努力奋斗！
